# Supplementary material for: Differential Lipidomics, Metabolomics and Immunological Analysis of Alcoholic and Non-Alcoholic Steatohepatitis in Mice
Source: Int J Mol Sci. 2023 Jun 19;24(12):10351. doi: 10.3390/ijms241210351 (PMC10299521; doi:10.3390/ijms241210351)
Supplement: Supplementary file 1 [file ijms-24-10351-s001.zip › supplemental data/supplemental data S1.pdf]

Figure S1

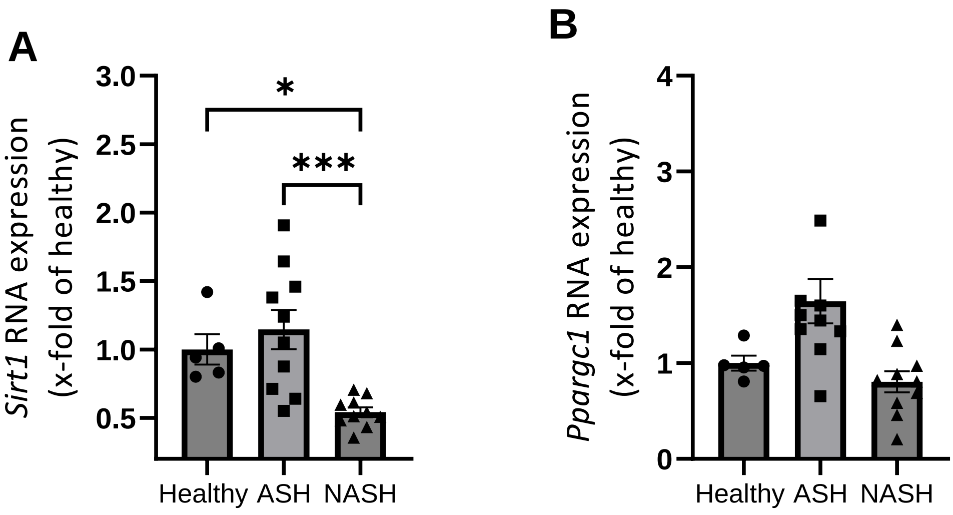

Figure S1: Expression of PGC-1α suggests a mild effect on mitochondrial functions in NASH (A,B) mRNA expression levels of SIRT1 (panel A) and PGC1a (panel B) in livers from health mice or mice with ASH and NASH. Data are shown as mean ± SEM, n = 4-10; One –way ANOVA Tukey post hoc test, *p<0.05; ***p<0.001
