# Supplementary material for: Differential Lipidomics, Metabolomics and Immunological Analysis of Alcoholic and Non-Alcoholic Steatohepatitis in Mice
Source: Int J Mol Sci. 2023 Jun 19;24(12):10351. doi: 10.3390/ijms241210351 (PMC10299521; doi:10.3390/ijms241210351)
Supplement: Supplementary file 1 [file ijms-24-10351-s001.zip › supplemental data/supplemental data S3.pdf]

Figure S3

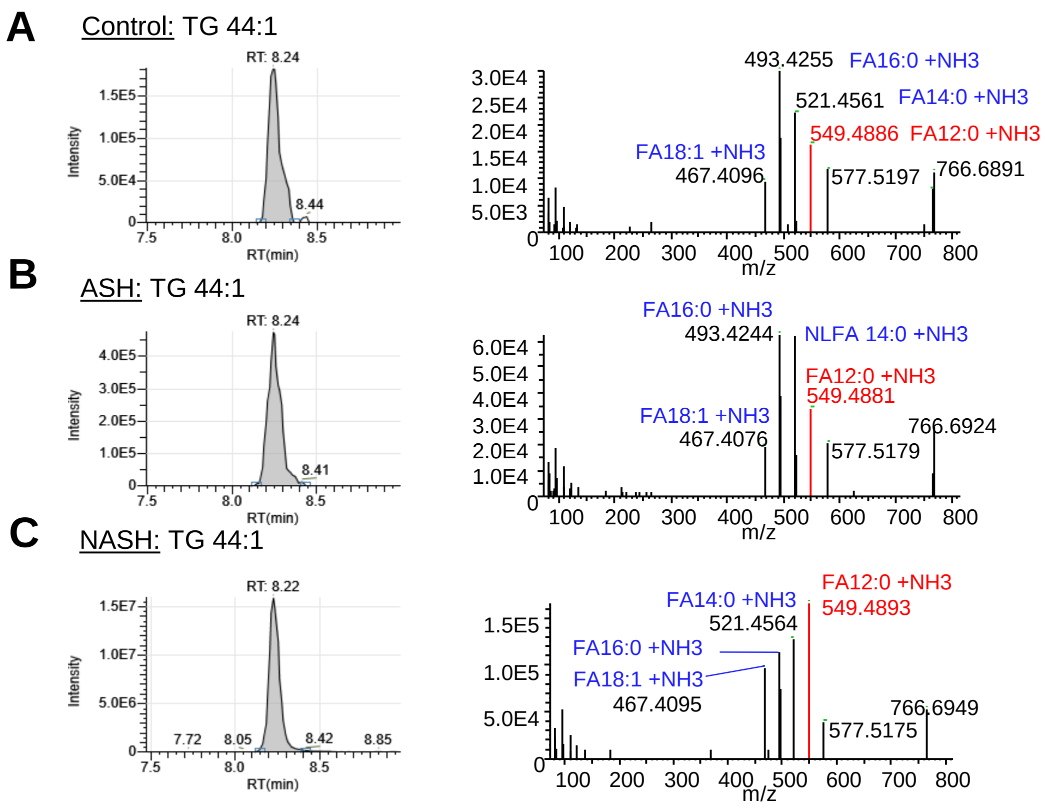

Figure S3: Diet-specific changes in lipidomics. **(A-C)** Representative chromatograms and MS2 spectra for TG 44:1 in livers from healthy mice (panel A), ASH (panel B) and NASH (panel C). m/z = 549.4893 and m/z = 521.4564 correspond to the fragments that underwent the neutral losses of FA 12:0 + NH3 and FA 14:0 + NH3, respectively (panel B). m/z = 199.1701 and m/z = 227.202 correspond to the hydrogen-loss fragments of FA 12:0 and FA 14:0, respectively (panels C, D). m/z signals corresponding to FA 12:0 and FA 14:0 are indicated in red and blue, respectively.
