# Supplementary material for: Differential Lipidomics, Metabolomics and Immunological Analysis of Alcoholic and Non-Alcoholic Steatohepatitis in Mice
Source: Int J Mol Sci. 2023 Jun 19;24(12):10351. doi: 10.3390/ijms241210351 (PMC10299521; doi:10.3390/ijms241210351)
Supplement: Supplementary file 1 [file ijms-24-10351-s001.zip › supplemental data/supplemental data S5.pdf]

Figure S5

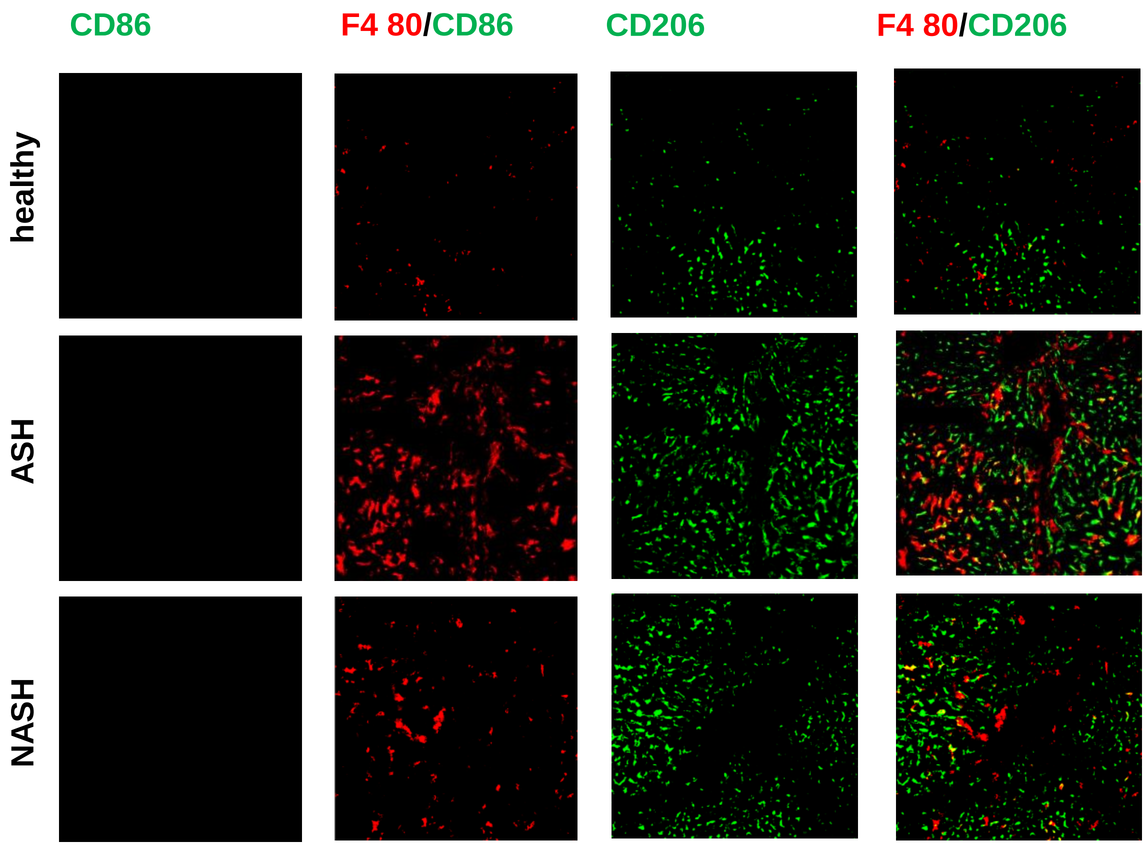

Figure S5: Representative images of M1- (F4 80^{+}/$CD86^{+}$) and M2-like macrophages (F4 80^{+}/$CD206^{+}$) in liver tissue 7 weeks after induction of ASH or NASH are shown.
