# Supplementary material for: Differential Lipidomics, Metabolomics and Immunological Analysis of Alcoholic and Non-Alcoholic Steatohepatitis in Mice
Source: Int J Mol Sci. 2023 Jun 19;24(12):10351. doi: 10.3390/ijms241210351 (PMC10299521; doi:10.3390/ijms241210351)
Supplement: Supplementary file 1 [file ijms-24-10351-s001.zip › supplemental data/supplemental data S6.pdf]

Table S6

Target	Company	Clone number	Order number
Arginase 1	Bioss	BS	bs-8585R
CD3	Miltenyi Biotec	REAG1	130-109-838
CD4	Southern Biotech	L3T4	1540-02
CD8	BD Pharmingen	53-6.7	553030
CD11b	BioRad	M1/70.15	MCA74F
CD11c	Miltenyi Biotec	N418	130-102-799
CD27	Miltenyi Biotec	REA499	130-114-166
CD29	Miltenyi Biotec	HMβ1-1	130-102-557
CD31	BD Biosciences	MEC13.3	553373
CD40	Biolegend	HM40-3	102905
CD41	AbDSerotec	MWReg30	MCA2245F
CD44	Miltenyi Biotec	REA664	130-119-121
CD45	Miltenyi Biotec	30F11.1	130-116-535
CD54	Biolegend	YN1/1.7.4	116105
CD80	Biolegend	16-10A1	104706
CD86	Biolegend	GL-1	105002
CD127	eBiosciences	A7R34	11-1271-82
CD206	Biolegend	C068C2	141708
CXCR3	R & D Systems	220803	FAB1685P
F4-80	Biolegend	BM8	123107
IFNg	eBioscience	XMG1.2	12-7311-41
IL1b	Thermo Fisher	NJTEN3	11-7114-82
IL-6	eBioscience	MP5-20F3	11-7061-82
IL-10	eBioscience	JES5-16E3	11-7101-41
Ki67	eBioscience	SolA15	11-5698-80
Ly6C	eBioscience	HK 1.4	17-5932-80
Ly6G	eBioscience	RB6-8C5	RM3005
MHC II	Miltenyi Biotec	REA813	130-112-233
Propidium Iodide	Sigma		P4170
Siglec F	BD Bioscience	E50-2440	552126
